# HPD: an online integrated human pathway database enabling systems biology studies

**DOI:** 10.1186/1471-2105-10-S11-S5

**Published:** 2009-10-08

**Authors:** Sudhir R Chowbina, Xiaogang Wu, Fan Zhang, Peter M Li, Ragini Pandey, Harini N Kasamsetty, Jake Y Chen

**Affiliations:** 1 Indiana University School of Informatics, Indianapolis, IN 46202, USA; 2 Indiana Center for Systems Biology and Personalized Medicine, Indiana University – Purdue University, Indianapolis, IN 46202, USA; 3 Department of Computer and Information Science, Purdue University School of Science, Indianapolis, IN 46202, USA

## Abstract

**Background:**

Pathway-oriented experimental and computational studies have led to a significant accumulation of biological knowledge concerning three major types of biological pathway events: molecular signaling events, gene regulation events, and metabolic reaction events. A pathway consists of a series of molecular pathway events that link molecular entities such as proteins, genes, and metabolites. There are approximately 300 biological pathway resources as of April 2009 according to the Pathguide database; however, these pathway databases generally have poor coverage or poor quality, and are difficult to integrate, due to syntactic-level and semantic-level data incompatibilities.

**Results:**

We developed the Human Pathway Database (HPD) by integrating heterogeneous human pathway data that are either curated at the NCI Pathway Interaction Database (PID), Reactome, BioCarta, KEGG or indexed from the Protein Lounge Web sites. Integration of pathway data at syntactic, semantic, and schematic levels was based on a unified pathway data model and data warehousing-based integration techniques. HPD provides a comprehensive online view that connects human proteins, genes, RNA transcripts, enzymes, signaling events, metabolic reaction events, and gene regulatory events. At the time of this writing HPD includes 999 human pathways and more than 59,341 human molecular entities. The HPD software provides both a user-friendly Web interface for online use and a robust relational database backend for advanced pathway querying. This pathway tool enables users to 1) search for human pathways from different resources by simply entering genes/proteins involved in pathways or words appearing in pathway names, 2) analyze pathway-protein association, 3) study pathway-pathway similarity, and 4) build integrated pathway networks. We demonstrated the usage and characteristics of the new HPD through three breast cancer case studies.

**Conclusion:**

HPD http://bio.informatics.iupui.edu/HPD is a new resource for searching, managing, and studying human biological pathways. Users of HPD can search against large collections of human biological pathways, compare related pathways and their molecular entity compositions, and build high-quality, expanded-scope disease pathway models. The current HPD software can help users address a wide range of pathway-related questions in human disease biology studies.

## Background

The study of biological pathways has become a central topic in molecular systems biology [1]. While the precise definition of "biological pathway" is still debatable, most researchers regard a biological pathway as a series of inter-connected cellular events among biomolecular entities. A biological pathway can be activated by extracellular stimuli and lead to persistent changes of the biochemical state of cells. There are three major types of molecular pathway events (or, *events *for brevity) that define biological pathways:

• **Signal transduction events**. Common in *signalling pathways *(e.g., Wnt signaling pathway [2]), these events define the interactions among molecular entities during signal transduction cascades, i.e., how external stimuli such as molecules in the cellular environment are transduced into intracellular molecular signals that are relayed among different cellular organelles. Examples of signal transduction events in signalling pathways are protein-protein interactions, protein post-translational modifications, protein translocations, and protein complex formations/dissociations.

• **Enzymatic reaction events**. Common in *metabolic pathways *(e.g., glycolysis pathway), these events define chemical reactions that metabolites (as either substrates or products) and catalytic enzymes are involved in. Examples of enzymatic reaction events are catabolic reactions (breaking down of larger molecules to produce energy) and anabolic reactions (synthesis of cellular components from smaller molecules).

• **Genetic regulation events**. Common in *genetic regulatory pathways *(e.g., usually abbreviated as *regulatory pathways*), these events define the dependent relationships between regulatory entities, e.g., a transcription factor that binds to specific DNA binding motifs, and target entities, and a gene whose transcription is being regulated by a transcription factor. In addition to gene regulation events, regulatory pathways may also include sRNA and sRNA target gene regulation.

Collecting and modeling biological pathways are critical for interpreting "Omics" data [3]. For example, pathway knowledge has been used to identify new functional modules from gene expression profiles [4,5] and relate gene mutations to one another in polygenic diseases such as breast cancer [6]. The development of biological pathways can also help build disease biology models, from which new hypotheses of targeted drugs and robust biomarkers may be developed. For example, molecular entities in FGFR1/PI3K/AKT signaling pathways, the Akt/PKB pathway, the Met pathway, and the Wnt signaling pathway have all been extensively investigated as potential cancer drug targets [7-10]. Novel drug discovery strategies to screen small molecules based on an entire pathway instead of particular protein targets can also be developed by designing global disease-related pathway inhibitors [11]. Pathway studies have also shown promise in molecular diagnostic applications, e.g., identifying efficacy and toxicity biomarkers [12], and building new multi-marker panels to improve prediction of disease prognosis and development of treatment plans [13]. Ongoing efforts to represent, develop, and apply pathway models will be crucial for future genome medicine and personalized medicine applications [14,15].

While there are approximately 300 biological pathway-related online resources reported by Pathguide http://www.pathguide.org/today, these resources have been developed with variable degrees of data coverage, quality, and utility [1]. Examples of high-quality biological pathway database resources are: SPAD [16], CST [17], STKE [18] and COPE [19] for signaling pathways; TRANSFAC [20] for regulatory pathways; and KEGG [21], WIT [22], ExPASy [23], UM-BBD [24] and HumanCyc [25] for metabolic pathways. In addition, new databases such as HPRD [26], HAPPI [27], and STRING [28] have been developed to provide available high-throughput protein-protein interaction data to help fill gaps in rapidly growing molecular signaling pathway data. Recent efforts to expand biological pathway coverage beyond a single pathway event type have also been reported, e.g., NCI-PID [29], Reactome [30], BioCarta [31], Pathway Commons [32], Panther [33], Protein Lounge [34] and WikiPathways [35]. However, by comparing the coverage of high-quality protein-protein interactions from the HAPPI database [27] with annotated human pathways documented from the Reactome database, for example, it is not difficult to conclude that current coverage of known human biological pathway events is 1–2 orders of magnitude smaller than the theoretical maximum that can be defined by all known reliable human protein-protein interactions. Therefore, many pathway biology studies begin by expanding biological pathway data coverage and building high-quality integrative pathway models.

The most reliable approach to expanding human pathway data coverage without sacrificing data quality continues to be database integration. While there are several computational techniques that can help predict metabolic pathways [36], regulatory pathways [37,38], and signaling pathways [39], they all have limited applicability and are thus beyond the scope of this work. However, integrating biological pathway from different data sources has been challenging, due to the heterogeneity in pathway data formats, representation schemes, and retrieval methods. For example, at the *syntactic *level, while many pathway databases such as the NCI-PID [29], Reactome [30], and KEGG [21] provide both molecular component and molecular interaction data as XML documents, Protein Lounge [34] and BioCarta [31] provide pathway details (including molecular entities and pathway events) only in TXT file and embedded pathway diagrams. Pathway ontology standards such as PSI-MI [40] or BioPAX [41] or GPML [42] can help resolve syntactic level data heterogeneity; however, these standards are relatively new and are available only in a few recent systems such as cPATH [43], NCI-PID [29], Reactome [30] and WikiPathways [35]. At the *semantic *level, incompatible pathway names, event representations, and molecular entity identifiers also poses challenges in querying pathway information across pathway data sources, particularly those with complementary information. Pathway names from different pathway data sources for the same pathway often differ slightly and therefore are poor choices as identifiers. Identifying pathways directly using pathway molecular entities can also be problematic, because the ensemble of molecular entities referring to the same pathway may vary among different annotation sources. Pathway molecular entities may be referred to with any public sequence identifier, which includes RefSeq ID, HGNC symbol, GenBank accession, SwissProt ID, UniProt name, KEGG ID, or IPI number. Furthermore, different databases may choose to provide available pathway information at different levels of molecular detail, e.g., with protein post-translational modification status, protein complex association status, or cellular location information. In summary, pathway data incompatibility at both the syntactic and semantic levels has inhibited the growth of high-quality integrative pathway data sources.

In this work, we describe the development of a new online integrated pathway database resource, the Human Pathway Database (HPD). HPD is an ongoing pathway data warehousing project, in which we integrate all three types of human pathway data and compile additional detailed information on pathway genes, proteins, metabolites, protein complexes, and pathway events. The concept of developing an organism-specific integrated pathway database resource is not unique, e.g., MAtDB [44] for managing all biological pathways for Arabidopsis and FlyMine [45] for managing both functional genomics and pathway data for *Drosophila*. Applying semantic-level data integration techniques, we collect, represent, and manage human-specific pathway data in HPD based on information from NCI-PID, Protein Lounge, KEGG, BioCarta, and Reactome databases. HPD provides a comprehensive view of current human biological pathway data, which consists of a total of 999 pathways and 59,341 molecular entities. Online HPD users may search the database for all relevant pathway information related to query protein(s), identify all pathways involving a query protein(s), and examine details related to pathway components, molecular events, and related pathways. Using three case studies, we show how to take advantage of HPD online and backend database querying capabilities to manage, query, and compare different types of biological pathways for systems biology studies. HPD is freely available online at http://bio.informatics.iupui.edu/HPD.

## Results

### Database content statistics

By integrating human biological pathway data from five major curated sources, we have developed HPD, a human pathway data warehouse. As of the current release, HPD contained a total of 999 human pathways that cover all three major types of pathway events. These pathways cover 59,341 molecular entities and 16,271 pathway events. As of April 2009, HPD contains the highest pathway data coverage among all human biological pathway databases publically available. Since HPD does not contain new pathways derived computationally, the quality of the database remains the same as that of each pathways curated from their respective source databases. A comparison of human pathways in HPD against several common human pathway data sources is shown in Table 1. Top 100 pathways, genes/proteins and compounds are listed in the additional file 1.

**Table 1:** A comparison of human pathways in HPD against several common pathway data sources.

		**BioCarta**^1^	**HumanCyc**^2^	**KEGG**^3^	**Reactome**^4^	**PID**^5^	**Protein Lounge**^6^	HPD
**Quality**	**Scope of Content**	Metabolic and signaling pathways	Metabolic Pathways	Metabolic, Regulatory, signaling, disease and drug pathways	Metabolic, signaling and regulatory pathways	Signaling and regulatory pathways	Metabolic, signaling and regulatory pathways	Metabolic, signaling and regulatory pathways
	
	**Curation Type**	Manual	Manual and Computational Prediction	Manual	Manual	Manual	Manual	Integrated from Manually curated database

**Coverage**	**Human Pathways**	354	327	205	960	87	427	999
	
	**Events**	> 3000	> 1500	4149	3203	5232	NA	16271

**Functionality**	**Related Pathways**	No	No	Yes	No	No	No	Yes
	
	**Multiple Protein Search**	No	No	No	No	Yes	No	Yes
	
	**Pathway-Protein Association Table**	No	No	No	No	No	No	Yes
	
	**Pathway-Pathway Similarity Network**	No	No	No	No	No	No	Yes

^1 ^Data from http://www.biocarta.com (as of April 2009)

^2^Data from http://humancyc.org/ (as of October 2008)

^3^Data from http://www.genome.jp/kegg/ (as of March 2009)

^4^Data from http://www.reactome.org (as of April 2009)

^5^Data from http://pid.nci.nih.gov/ (as of April 2009)

^6^Data from http://www.proteinlounge.com (Commercial Version, as of April 2009)

** The count of HPD pathways has 39 pathways more than reactome since HPD utilizes 2004 Reactome (version 22 release). The next version would include the recent version from Reactome

### Scale distributions of integrated HPD pathways

**Figure 1:**
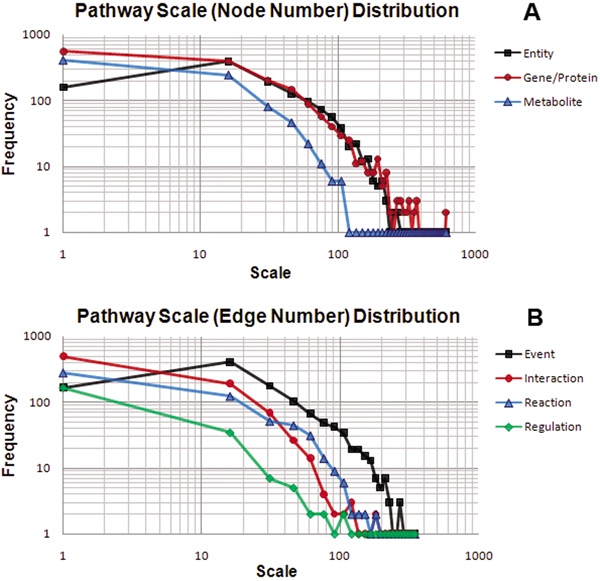
**Pathway scale distributions for HPD molecular entity data and molecular event data**. A pathway scale refers to the number of molecular entities or molecular events involved in a given pathway. The frequency on the y-axis refers to the count of all pathways falling in the category of a particular pathway scale size on the x-axis. (a) Distributions of pathway scale by counting molecular entities. (b) Distributions of pathway scale by counting molecular events.

Pathway scale can reflect the integrality of information needed for a biological topic. Here, we define pathway scale as the number of entities (nodes, including gene, protein, complex and metabolite) or events (edges, including interaction, reaction and regulation) involved in a pathway. We performed a statistical analysis on Pathway Scale Distribution (PSD) in the whole HPD, shown in Figure 1, from which we can see that the PSD defined by entity in Figure 1a is almost the same as the PSD defined by event in Figure 1b. This result indicates that the ratio of entity (node) number and event (edge) number in HPD pathways is almost fixed, which implies that the quality of HPD is consistent. We can also find that the PSD defined by gene/protein in Figure 1a is much closer to the PSD defined by entity or event than the PSD defined by metabolite, interaction, reaction and regulation, which suggest that using gene/protein number can represent pathway scale more precisely. This is the most important evidence not only for the implemental definition of pathway scale, but also for the definition of pathway-pathway similarity, both of which can be defined by the number of the Uniprot IDs mapped from genes or proteins in a pathway.

**Figure 2:**
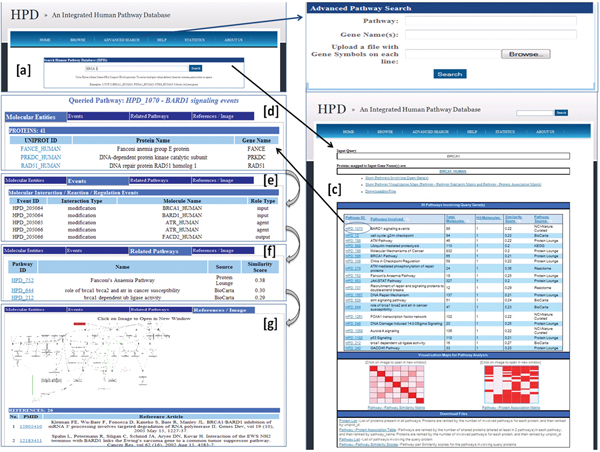
**An overview core functionality of the online HPD software**. (a) The HPD home page showing a query protein input box (supports multiple protein search using either gene names or UniProt identifiers). (b) A Web page containing the list of pathways retrieved as a result of a query protein input. Links to obtain pathway-pathway similarity matrix and pathway-protein association matrix and to download query-retrieved pathways were also shown on the Web page. (c) An advanced search page in which users may search pathways using gene name(s), with any term that appears within pathway names or with a list of one or more HPD Pathway IDs. This search retrieves a page similar to in (b). (d) A hyperlinked Web page showing pathway detailed information on molecular entities (Proteins, Complexes and Compounds) within the query pathway. (e) A hyperlinked Web page showing detailed pathway information on pathways similar to the query pathway. (f) A hyperlinked Web page showing detailed pathway information on molecular events (Interaction/Reaction/Regulation) within the query pathway. (g) A hyperlinked Web page showing pathway image link and reference articles.

We can also notice that, since entities in a pathway here also include protein complexes, each of which will only count as one entity, the PSD defined by that entity is a little bit lower than the PSD defined by gene/protein in Figure 1a. Both of the results in Figure 1a and 2b suggest that the integration process of HPD is successful by considering pathway scales, but either small pathways or large pathways may still be under-represented in the whole HPD.

### General online features

**Figure 3:**
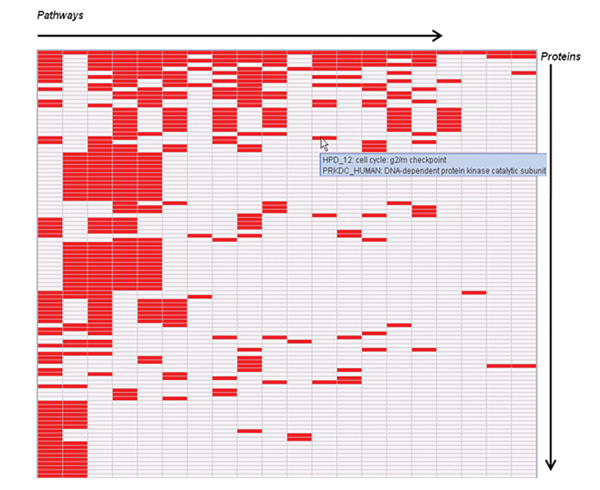
**A BRCA1-retrieved HPD pathway-protein association matrix**. The matrix shows few HPD pathways involving the query protein BRCA1 (on the y-axis) against pathway molecular components (on the x-axis). A red cell in the matrix indicate that the molecular entity is present in the pathway, whereas a white cell in the matrix indicate that the molecular entity is absent from the pathway. Only few HPD pathways are shown. These BRCA1-retrieved pathways are sorted by their shared protein counts among all pairwise pathway comparisons in these pathways.

**Figure 4:**
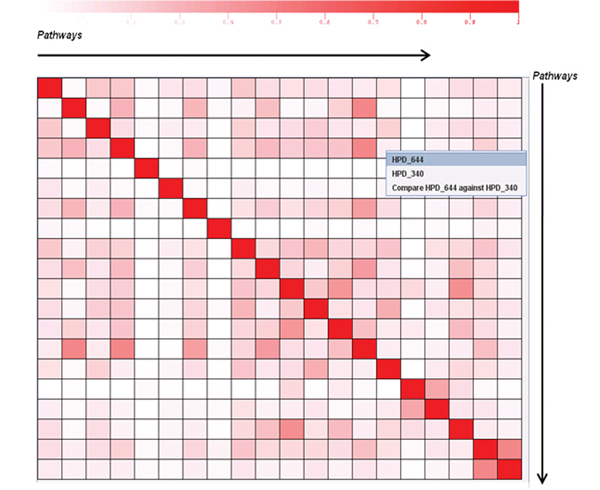
**A BRCA1-retrieved HPD pathway-pathway similarity matrix**. This is an interactive heat map containing similarity scores among the pathways involving query protein(s) or gene name(s). The tooltip shows the two pathway IDs and names corresponding to the particular cell pointed to along with their similarity score. A right click context menu shows the links (HPD_644 and HPD_340) to a Web page containing pathway information (as shown in Figure 2d). The "Compare HPD_644 against HPD_340" option will redirect to a page with a pathway-protein matrix showing proteins shared by these two pathways. The legend above the map indicates the range of similarity score (0 to 1).

In Figure 2, we show the user interfaces of the Web-based online version of HPD. It supports both standard and customized user search options that allow them to specify a list of genes/proteins or keywords as the query input. Upon executing the queries, HPD can retrieve a list of related human pathways in an HTML table, with which users can further explore pathway details by clicking the hyperlink on a pathway ID in the table. In the pathway detail HTML table that pops up, all listings of molecular entities, events, related pathways, and reference resources of a specific pathway are shown. Users can also directly interact with advanced HPD features; by selecting the pathway-protein association matrix applet (See Figure 3 for an example) and the pathway-pathway similarity matrix applet (See Figure 4 as an example) Comprehensive hyperlinks were built so that users can search for new pathways based on visual analysis performed on the applets. User queried pathway data stored in HPD can also be downloaded as flat files without restriction to Academic users.

### Case studies

To demonstrate the capabilities of HPD, we show three case studies of increasing complexity and biological significance to demonstrate how HPD could be used to solve real-world biological pathway problems.

### Case study 1: searching for biological pathways and their components based on a single query protein

Using the standard query box provided at the HPD home page, we can search HPD for all biological pathways involving *BRCA1_HUMAN *(a major protein involved with breast cancer susceptibility). HPD returns a list of the top 20 BRCA1-related pathways, which are ordered by decreasing number of proteins that each pathway shares among all pathway pairs from retrieved pathways. The better the rank a retrieved pathway has, the more related it should be to both the query protein BRCA1 and all BRCA1-relevant pathways. In this list, highly-ranked pathways such as "Molecular Mechanisms of Cancer", "P53 Signaling", "DNA Repair Mechanism", and "BRCA1 pathway" are all well characterized signaling pathways in breast cancer. All pathways are hyperlinked to their own detailed pathway information pages, which include molecular entities (proteins, complexes and metabolites), related pathways, events, and external pathway images and reference articles. (See Figure 2 for details).

The Web page with the list of pathways related to BRCA1 also contains links to download data. Four types of data, pathway list, pathway-protein association matrix, and pathway-pathway similarity scores are downloadable as flat files.

Note that the pathway-protein association matrix contains proteins that are involved in the top 20 pathways retrieved based on the single protein query, sorted according to their descending maximal pathway involvement by activity count. BRCA1 related proteins are retrieved by pathway, with each of the proteins covered by at least two of the 20 pathways. A close examination reveals that many breast cancer susceptibility genes including *BRCA1, BRCA2, P53, PCNA *[46], *FOXA1 *[47] and *STK6 *[48] from recent individual studies and breast cancer biomarker genes such as *ERBB2, FGFR2, M3K1, and PTEN *[49,50], have all been found in this list.

Particularly noteworthy is the Applet in the HPD Web page that shows all the query-related biological pathways with involved proteins in a heat map. In Figure 3, *BRCA1 *related pathways and involved proteins are sorted and used as two separate dimensions of the matrix. Mousing over a color-filled cell invokes an applet tooltip message, which shows the pathway and protein names.

HPD users can also visualize the pathway-pathway similarity matrix (Figure 4) which shows the similarity score among the *BRCA1 *related pathways. The pathway-pathway similarity matrix allows users to visualize a cluster of similar pathway pairs as a 2-D interactive heat map. This heat map allows users to right click on any cell (shown in Figure 4) to compare pathway pair on the heat map (future versions will include multiple pathway selection) by looking at the pathway-protein association matrix. This facilitates better understanding for deriving novel pathways most similar to *BRCA1 *related pathways.

### Case study 2: developing pathway-pathway similarity networks from heterogeneous data sources

Using the advanced HPD search function online, a user can specify multiple proteins as the query input to obtain a list of most relevant pathways related to the query protein set. For example, if the user enters "BRCA1_HUMAN, FOXA1_HUMAN, STK6_HUMAN" as query inputs, a significant number of pathways (Table 2) related to any of the query protein inputs will be returned. To ensure retrieved pathways are relevant to the query protein inputs and to avoid overly restrictive filtering of related pathways (e.g., requiring all pathways retrieved to contain all proteins in the input query would be too restrictive), we can use the concept of pathway similarity (see Methods section for details) and apply a minimal pathway similarity threshold {*S*_*i*, *j *_≥ 0.2, and |*P*_*i *_∩ *P*_*j*_| > 2}, *i *= 1...*N*, *j *= 1...*N*. The threshold indicates at least 20% minimal shared molecular entities with no fewer than 2 shared entities between two pathways. After applying this filter, 25 pathways and 39 pathway pairs are retrieved.

**Table 2:** A list of HPD pathways retrieved by the query BRCA1.

Pathway_ID	Pathway_Name	Source_Name	Shared_Proteins
**HPD_786**	Molecular Mechanisms of Cancer	Protein Lounge	264
**HPD_953**	JAK/STAT Pathway	Protein Lounge	239
**HPD_1182**	p53 Signaling	Protein Lounge	76
**HPD_1567**	DNA Repair Mechanism	Protein Lounge	69
**HPD_336**	Chks in Checkpoint Regulation	Protein Lounge	52
**HPD_596**	BRCA1 Pathway	Protein Lounge	44
**HPD_1058**	Aurora A signaling	NCI-Nature Curated	43
**HPD_1070**	BARD1 signaling events	NCI-Nature Curated	40
**HPD_788**	ATM Pathway	Protein Lounge	33
**HPD_644**	role of brca1 brca2 and atr in cancer susceptibility	BioCarta	27
**HPD_988**	Ubiquitin mediated proteolysis	KEGG	25
**HPD_12**	cell cycle: g2/m checkpoint	BioCarta	23
**HPD_628**	atm signaling pathway	BioCarta	20
**HPD_340**	GADD45 Pathway	Protein Lounge	19
**HPD_752**	Fanconi's Anaemia Pathway	Protein Lounge	17
**HPD_548**	DNA Damage Induced 14-3-3Sigma Signaling	Protein Lounge	14
**HPD_212**	brca1 dependent ub ligase activity	BioCarta	11
**HPD_1261**	FOXA1 transcription factor network	NCI-Nature Curated	10
**HPD_707**	Recruitment of repair and signaling proteins to double-strand breaks	Reactome	9
**HPD_276**	ATM mediated phosphorylation of repair proteins	Reactome	5

**Figure 5:**
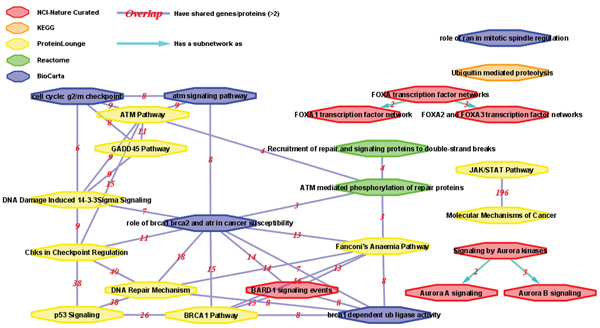
**A breast cancer-specific pathway-pathway similarity network**. In this pathway-pathway similarity network, 25 HPD pathways derived from different sources are shown. The color and shapes in the diagram were drawn to indicate original HPD pathway data sources, based on the shape/color legend shown in the upper left corner. The subnetwork pathways and host pathways are also indicated with directed cyan edges. Edges are labeled with the number (in red) of molecular entities shared by the connected pathways. The count of molecular entity overlap between each pair of related pathways is labeled as red-colored numbers on the edge. Only pathway pairs with a similarity score and overlap above the threshold {*S*_*i*, *j *_≥ 0.2, AND |*P*_*i *_∩ *P*_*j*_| >2} are shown.

In Figure 5, we show a visual display of the pathway-pathway similarity network, using pathway similarity scores retrieved from HPD using *ProteoLens *[51]. In order to generate a comprehensive perspective of breast cancer pathways seeded with the three initial query proteins, all five types of data sources have been used. This observation strengthens the claim for the necessity of integrating pathways from heterogeneous sources. HPD pathways in this case study provide a good meta-model that connects our fragmented pathway knowledge together in pathway-pathway similarity networks. This global perspective, supported by integration of otherwise incompatible pathways from different sources, enhances the chance of exposing novel insights in the search for disease drug targets and biomarkers.

**Figure 6:**
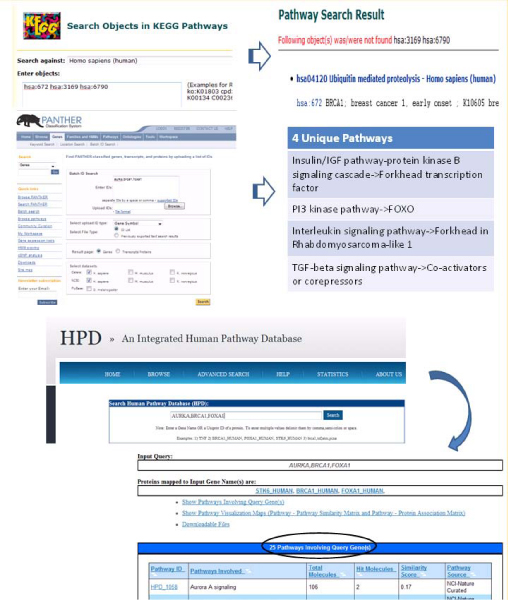
**A comparison of HPD "Multiple Protein Search" feature with those in Panther and KEGG**. The example shows that different pathway databases retrieved different numbers of pathways. Three genes (AURKA, BRCA1, and FOXA1) were used to build this example. The KEGG database returned only one pathway in the result. Panther returned four pathways. HPD returned 25 pathways. The difference is primarily due to the use of "similar pathways" concept to allow retrieval of pathways that matched only partial gene/protein list.

In Figure 6, we show a comparison of using the "Multiple Protein Search" feature among three databases: HPD, KEGG, and Panther. Three gene names BRCA1, FOXA1, and AURKA were used to build a common query gene set. The KEGG Genes database was manually searched and only one KEGG Pathway was found, using the "Search object in Pathways" functionality of KEGG (actual corresponding KEGG gene ID entered: hsa:672, hsa:3169, and hsa:6790). Panther had a "Batch ID search" which accepted the three gene symbols and retrieved only four unique pathways. HPD not only retrieved more pathways (n = 25), but also supported multiple identifier types as inputs, e.g., UniProt names.

### Case study 3: developing integrated pathway models from heterogeneous sources

While pathway-pathway similarity networks are useful for generating global perspectives on the relationships between pathways, the next case study demonstrates how to connect different types of biological pathways within HPD to form integrated pathway networks. Since pathway data managed at HPD is integrated at the schematic level, "deep integration" and "deep integrative analysis" are possible. We will use two breast cancer-related proteins, BRCA1_HUMAN and FOXA1_HUMAN, as an example. According to the HPD data model (See additional file 2 for details), the table *Connect_mol_updated *contains mappings among pathways, interactions, and molecules. To search for all related pathways containing the above two proteins within the HPD data warehouse, we can execute the following SQL query:

SELECT pathway_name,mol_in, Mol_In_updated, name_in, Mol_out,

Mol_Out_updated, name_out, interaction_type, SYS_CONNECT_BY_PATH(Mol_In, '/') "Path"

FROM connect_mol_updated

START WITH name_in = 'BRCA1_HUMAN'

CONNECT BY nocycle PRIOR Mol_Out_updated=Mol_In_updated

and level < 3

INTERSECT

SELECT pathway_name,mol_in, Mol_In_updated, name_in, Mol_out,

Mol_Out_updated, name_out, interaction_type, SYS_CONNECT_BY_PATH(Mol_In, '/') "Path"

FROM connect_mol_updated

START WITH name_in = 'FOXA1_HUMAN'

**Figure 7:**
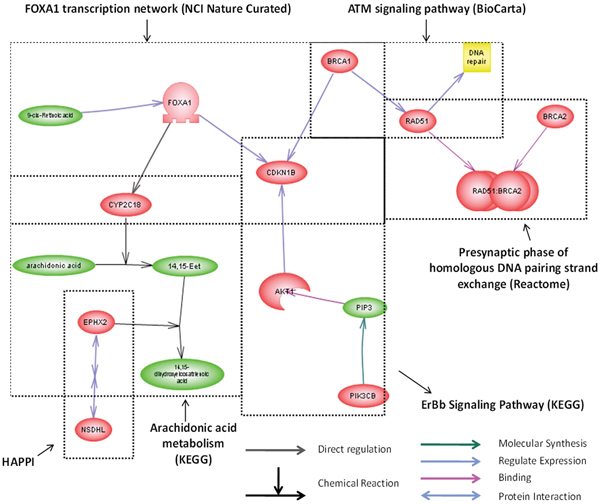
**An integrated pathway model involving FOXA1 in breast cancer**. The figure shows how information from different pathway database sources are readily integrated, queried, and analyzed together in HPD for FOXA1-related breast cancer signaling studies.

CONNECT BY nocycle PRIOR Mol_Out_updated=Mol_In_updated

and level < 3;

We organize the results and present our final pathway analysis results in Figure 7, which shows many relationships not found in individual fragmented biological pathways separately. The *FOXA1 *transcription factor network contains 9-cis-Retinoic acid which regulates *FOXA1 *(Hepatocyte nuclear factor 3-alpha) [52]; it also contains *BRCA1 *(Breast cancer type 1 susceptibility protein) and *CYP2C18 *(Cytochrome P450 2C18), which is positively regulated by *FOXA1 *[53]. Arachidonic acid metabolism from KEGG PATHWAY involves arachidonic acid, which can be catalyzed by *CYP2C18 *[54] to produce 14,15-epoxy-5,8,11-eicosatrienoic acid. This intermediate product can be further catalyzed by *EPHX2 *(Epoxide hydrolase 2) to produce 14,15-dihydroxyeicosatrienoic acid [55]. In BioCarta, ATM signaling pathway involves *BRCA1*, which positively regulates *RAD51 *which regulates DNA Repair [56]. The "Presynaptic phase of homologous DNA pairing and the strand exchange" pathway of Reactome contains *BRCA2*, which binds with *RAD51 *to form the *RAD51:BRCA2 *complex [57]. Human protein-protein interactions data could also be retrieved and show that *EPHX2 *interacts with *NSDHL *(Sterol-4-alpha-carboxylate 3-dehydrogenase, decarboxylating). Phosphatidylinositol-3,4,5-triphosphate (PIP3), a lipid molecule generated by the action of phosphoinositide-3-kinase (*PI3K*), can be induced by a variety of stimuli. PIP3 is thought to be the major physiological substrate for *PTEN*, a phosphatase that can dephosphorylate many phosphatidyl inositides, which has been implicated in tumorigenesis [58]. Activation of protein kinase B *(PKB)/Akt *contribute to resistance to antiproliferative signals and breast cancer progression in part by impairing the nuclear import and action of *p27 (CDKN) *[59].

The integrated pathway model based on HPD pathways can be used as an investigative tool for disease diagnostic and therapeutic applications. For example, 9-cis-Retinoic acid is recognized as a possible breast cancer biomarker [60] and *FOXA1 *has gained increasing attention as a possible breast cancer therapeutic target [61]. The *BRCA2-RAD51 *interaction is essential for DNA repairs and has also been suggested as a novel target for anti-breast cancer drugs [62]. In addition to breast cancer, links between breast cancer and other diseases can be studied. For example, increased risk of hereditary prostate cancer is known to be a result of polymorphism in the *CDKN1B (p27) *gene [63]. Epoxide hydrolase 2 has been characterized as a key mediator molecule in hypertensive, cardiovascular, inflammatory, pulmonary, and diabetic-related diseases [64-66]. CHILD syndrome, an X-linked dominant trait with lethality for male embryos, can also be traced to mutations in *NSDHL*, a gene playing crucial roles in the cholesterol biosynthetic pathway [67].

Through this case study, we have shown the significance of integrating pathway information from different types and data sources. The interconnected network analysis offers researchers a rare opportunity to gain global perspectives on events previously perceived in isolation. This "deep integrative analysis" opportunity cannot be readily obtained by using multiple online pathway databases. For example, NCI Nature Curated Pathway Interaction Database has a 'Connected Molecules' functionality, which may only be used to find molecular connections within the same pathway data source. In all, the convenience of building new integrative pathway models with the new HPD may greatly facilitate new drug development and biomarker discovery.

## Conclusion

We developed HPD as an integrated pathway database system to manage, query, and analyze human biological pathways. HPD integrates all three types of biological pathways from five heterogeneous pathway database sources at syntactic, semantic, and schematic levels, primarily based on data warehousing techniques driven by a unified pathway data model. Pathway molecules, interactions, chemical reactions, and similar pathways can be searched, displayed, and downloaded from a unified online user interface. The current HPD software can help users address a wide range of pathway-related questions in human disease biology studies.

While the human Reactome is still far from complete, an integrative pathway database such as HPD has the capability to help researchers establish a global perspective necessary for understanding molecular mechanisms and develop biomedical applications. We will further expand the database to include pathways from HumanCyc [25], Wikipathways [35], NetPath [68], Panther [33] and TRANSFAC [20]. We also plan to integrate protein-protein interaction data from HAPPI [27] with the aim of discovering novel pathways when combined with HPD. Additional functions will also be provided such as pathway reconstruction where users can select pathways and derive a reconstructed pathway expanded with protein-protein interaction data. With ongoing efforts, HPD can become a useful resource, linking proteins, genes, RNAs, signaling reactions, and gene regulatory events for systems biology applications.

## Methods

### Pathway data sources

**Figure 8:**
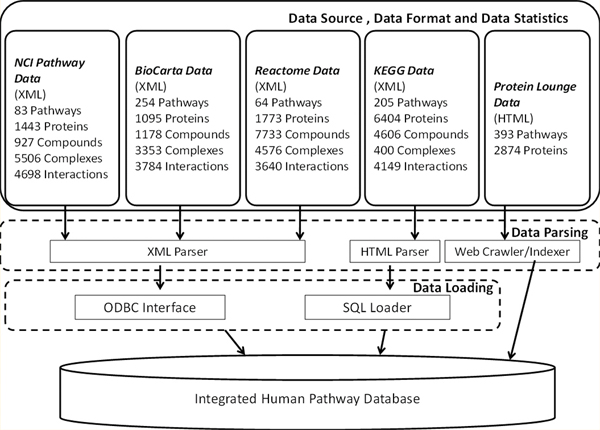
**An overview of pathway data integration process**. The figure shows the whole process of pathway data integration and the basic statistics of pathway data sources.

We show an overview of the data integration process in Figure 8. Pathway data in HPD were collected or indexed from five different sources, i.e., NCI-Nature Curated data [29], BioCarta [31], Protein Lounge [34], Reactome [30] and KEGG [21]. The NCI-Nature Curated, Reactome and BioCarta data sets were all downloaded from Nature pathway interaction database Website and kept updated as of April 2009 release of the production HPD Website. In particular, the NCI-Nature Curated pathways are curated by Nature Publishing Group editors based on known biomolecular interactions and key cellular processes of signaling/regulatory pathways. The Reactome database was downloaded in December 2007 (Release version 22). Pathway molecules from both NCI-Nature and Reactome were identified by their UniProt identifiers and annotated with post-translational modification information. Pathway molecules from BioCarta were identified by Entrez Gene IDs without post-translational modification annotations. In all three data sets, each pathway was represented as a series of events, each of which consists of molecules in one of the following four roles: input molecule, output molecule, agent, and inhibitor. Content from the Protein Lounge was indexed by a web crawler accessing a publicly available Web site. The crawled content was verified with that provided as a site license to the authors and other users at Indiana University Simon Cancer Center. Since Protein Lounge is a commercial database that contains curated signaling, transduction, and metabolic pathways, we chose to index instead of integrate the full content into the data warehouse, which only indexed pathway-involving protein IDs and references to pathway diagram drawings. Original pathway molecules derived from Protein Lounge were identified by RefSeq ID or GI accession numbers. KEGG contains all known metabolic pathways and a small number of regulatory pathways and transport mechanisms. The KEGG PATHWAY database contains graphical representations of pathways and lists of enzymes and reactions within the pathways. All specific pathway maps and overviews were manually drawn and contained links to additional information on pathway compounds, enzymes and genes. Pathway molecules from KEGG are identified by E.C. Numbers which are mapped to KEGG Gene IDs and then to UniProt IDs. The total count of initial pathways, proteins, compounds, protein complexes, and pathway interactions are shown in Figure 8.

### Pathway data integration

We developed a model-driven approach for syntactic, semantic, and schematic level integrations of heterogeneous pathway data. Since pathway data were collected in a variety of formats, Python XML/HTML data parsers were developed to convert them into a common tab-delimited textual format to ensure syntactic level data compatibility. The semantic compatibility of the data was enforced by cleaning up data attributes and data values to keep them consistent, using a standard data extraction, transformation, and loading (ETL) process characteristics of data warehousing-based data integration approaches. All pre-processed data were parsed, cleaned, and loaded into data warehouse staging tables before reaching their final database table destinations. To maintain schematic data compatibilities, we model relationships among different pathway concepts using an entity-relationship (ER) data model (for more details on the data model, please refer to the documentation on the HPD Website and additional file 2). We further mapped all the involved proteins or genes to their UniProt Name Identifiers [69] and metabolites to their KEGG compound IDs before loading the HPD pathway data into data warehouse tables defined by the ER data model. All HPD molecular entities, events, and pathways were assigned unique HPD-specific identifiers.

### Online HPD software design

The HPD database was developed as a data warehouse application. The online version of HPD is a standard 3-tier Web application, which consists of an Oracle 10 g database at the backend database server layer, Apache/PHP server scripts at the middleware application Web server layer, and CSS-driven Web pages presented at the browser.

### Pathway similarity measure

The pathway similarity measure can be defined as the extent of overlaps, e.g., common number of genes/proteins, shared between two different pathways. We define a pathway-pathway similarity score *S*_*i*, *j *_based on equation (2) [70]. Both overlap and similarity score values can be downloaded from HPD Website.(2)

Here, *N *denotes total number of pathways. *P*_*i *_and *P*_*j *_denote two different pathways, while |*P*_*i*_| and |*P*_*j*_| are the numbers of molecules that can be mapped to UniProt ID respectively in these two pathways. Their intersection *P*_*i *_∩ *P*_*j *_denotes a common set of molecules that can be mapped to the same UniProt ID, while their union *P*_*i *_∪ *P*_*j *_is calculated as |*P*_*i*_| + |*P*_*j*_| - |*P*_*i *_∩ *P*_*j*_|. Here *α *is a weight coefficient among [0, 1], and we currently use *α *= 0.8 to count varying degree of contributions from calculations based both on the *overlap *(left item *S*_*L*_) and the *cover *(right item *S*_*R*_).

We can also make special considerations for *subnetwork relationship *(defined by the Nature Pathway Interaction database at http://pid.nci.nih.gov/. For subnetwork relationship, we define *S*_*i*, *j *_= 1.01, if pathway *P*_*i *_has a subnetwork as *P*_*j*_, and *S*_*i*, *j *_= -1.01 if pathway *P*_*i *_is a subnetwork of *P*_*j*_.

## Competing interests

The authors declare that they have no competing interests.

## Authors' contributions

JYC conceived the initial work, designed the method for the database construction, and drafted the manuscript. SRC and HNK implemented the design, and developed the database from integrated data sets. SRC, FZ and PML implemented the Web-based database interface. XW performed HPD pathway analysis to generate the first two case studies and statistical analysis. RP and SRC together implemented data warehousing strategies, provided ID mapping tables, and performed data processing, extraction, transformation, and loading. All authors are involved in the revisions of the manuscript.

## Supplementary Material

Additional file 1 This additional file lists top 100 pathways ranked by degree (number of neighbour pathways, with which similarity score > 0); top 100 genes/proteins ranked by frequency, and top 100 compounds ranked by frequency. Here the frequency of a molecule entity (i.e. gene/protein or compound) also includes times appearing in same pathways. Click here for file

Additional file 2 This additional file describes the pathway entity-relationship (ER) data model for HPD pathway integrations in detail. Click here for file
