# Supplementary material for: HPD: an online integrated human pathway database enabling systems biology studies
Source: BMC Bioinformatics. 2009 Oct 8;10(Suppl 11):S5. doi: 10.1186/1471-2105-10-S11-S5 (PMC3226194; doi:10.1186/1471-2105-10-S11-S5)
Supplement: Additional file 1 — This additional file lists top 100 pathways ranked by degree (number of neighbour pathways, with which similarity score > 0); top 100 genes/proteins ranked by frequency, and top 100 compounds ranked by frequency. Here the frequency of a molecule entity (i.e. gene/protein or compound) also includes times appearing in same pathways. [file 1471-2105-10-S11-S5-S1.doc]

## A pathway entity-relationship (ER) data model for HPD pathway integrations

Based on our past experience with pathway data and lessons learned from earlier version of HPD, we developed a pathway entity-relationship (ER) data model for HPD. This data model unifies the representation of all integrated pathway entities, including molecules, complexes, compounds, regulatory relationships of molecules, and reactions involved in metabolic, signaling and regulatory pathways. HPD uses three types of events: Interaction (protein-protein interactions) represented more in signaling pathways, Reaction (protein-compound interactions) in metabolic pathways and Regulation (protein-gene interaction) in gene regulatory pathway.

This integrated data model allows easy representation and data management on various types of pathway components; such as m-to-m relationships among “Molecular Interactions”, multiple names and alias from different database sources for each HPD molecule.


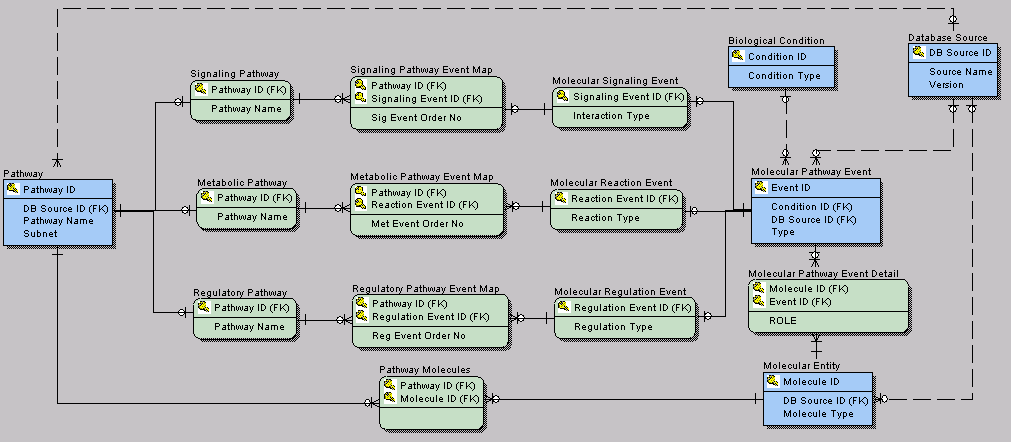


## Figure S1. The domain data model for HPD pathway integrations.
